# Real world evidence of adjuvant trastuzumab in HER2 positive early breast cancer

**DOI:** 10.1038/s41598-023-34429-9

**Published:** 2023-05-03

**Authors:** J. Lluch-Gómez, V. Núñez-Álvarez, C. de la Torre-Hita, M. Bernal-Gómez, A. Campini-Bermejo, E. Perdomo-Zaldívar, L. Rodríguez-Pérez, J. Calvete-Candenas, M. J. Martínez-Bautista, E. Benítez-Rodríguez, J. M. Baena-Cañada

**Affiliations:** 1 Medical Oncology Department, Hospital Universitario Puerta del Mar (HUPM), Ana de Viya 21, 11009 Cádiz, Spain; 2 Instituto de Investigación e Innovación Biomédica de Cádiz (INIBiCA) [Institute for Biomedica Research and Innovation], Ana de Viya 21, 11009 Cádiz, Spain; 3 Pharmacy Unit, Hospital Universitario Puerta del Mar (HUPM), Avenida de Ana de Viya 21, 11009 Cádiz, Spain; 4 Preventive Medicine Department, Hospital Universitario Puerta del Mar, Avenida de Ana de Viya 21, 11009 Cádiz, Spain

**Keywords:** Cancer, Breast cancer, Oncology

## Abstract

Adjuvant trastuzumab in HER2+ breast cancer reduces recurrence and mortality, and has been the standard treatment since 2006. The objective was to analyze health outcomes in the real world. Observational, retrospective study of patients with HER2+ breast cancer, stages I–III, treated with adjuvant trastuzumab in the past 15 years in only one center and for the first time in Spain. Survival was analyzed according to the number of cycles and cardiotoxicity. Two hundred and seventy-five HER2positive patients (18.60%) out of 1479 received adjuvant (73%) or neoadjuvant/adjuvant (26%) trastuzumab, concomitantly (90%) or sequentially (10%) with chemotherapy. The probability of overall and disease-free survival (OS and DFS) at 5 years was 0.93 (95% CI 0.89–0.96), and 0.88 (95% CI 0.83–0.92). The number of cases with a significant and asymptomatic decrease in ventricular ejection fraction and heart failure were 54 (19.64%) and 12 (4.36%), respectively. Sixty-eight patients (24.70%) received 16 or fewer cycles, especially those older than 65 (OR 0.371, 95% CI 0.152–0.903; p = 0.029) and with cardiotoxicity (OR 15.02, 95% CI 7.437–30.335; p < 0.001). The risk of cardiotoxicity was associated with having received radiotherapy (OR 0.0362, 95% CI 0.139–0.938; p = 0.037). Arterial hypertension (HR 0.361, 95% CI 0.151–0.863, p = 0.022), neoadjuvant treatment (HR 0.314, 95% CI 0.132–0.750, p = 0.009) and cardiotoxicity (HR 2.755, 95% CI 1.235–6.143, p = 0.013) maintained significant association with OS. Only neoadjuvant treatment maintained a significant association with DFS (HR 0.437, 95% CI 0.213–0.899, p = 0.024). The effectiveness of neoadjuvant and adjuvant trastuzumab can be considered comparable to those of clinical trials. In the real world, factors such as age, hypertension, radiotherapy, neoadjuvant treatment, and cardiotoxicity should be taken into consideration to optimize outcomes.

## Introduction

HER2 (*Human epidermal growth factor receptor 2*) is a member of the family of four transmembrane receptor tyrosine kinases that regulate cell growth, survival, and differentiation through multiple signal transduction pathways^1^. Of all patients with breast carcinoma, the HER2-positive subtype overexpresses this receptor, it accounts for 20% of cases^2,3^ and is associated with a worse prognosis^4^.

Trastuzumab, a monoclonal antibody directed against the HER2 protein extracellular domain, is active in patients with HER2-positive breast cancer^5^. Targeted therapy with 1 year of trastuzumab in combination with chemotherapy has been shown to significantly improve disease-free survival (DFS) and overall survival (OS) in HER2-positive early stage breast cancer^6–8^. Several trials have investigated shorter durations of adjuvant trastuzumab, between 9 weeks and 6 months, with varying results^9–13^. Systematic review of these trials finds that, compared with 1 year, the shorter duration of adjuvant trastuzumab is associated with significantly worse DFS and OS, despite the more favorable cardiotoxicity profile^14^.

The cardiac toxicity associated with trastuzumab is primarily heart failure and is a significant adverse effect because it prevents patients from receiving the full treatment and ultimately affects outcomes and survival^15^. It ranges from asymptomatic decrease in left ventricular ejection fraction (LVEF) to overt congestive heart failure, and is often reversible^15^. It is important to assess baseline risk factors in all patients and regular monitoring of LVEF using echocardiography or isotopic ventriculography during the year of adjuvant treatment^16^.

This study analyzed the outcomes of women with HER2-type breast carcinoma who received adjuvant trastuzumab since its approval by the European Medicines Agency (https://www.ema.europa.eu/en/medicines/human/EPAR/herceptin#assessment-history-section) and by the Spanish Agency of Medicines and Medical Devices (*Agencia Española de Medicamentos y Productos Sanitarios*) (https://www.aemps.gob.es/informa/notasInformativas/medicamentosUsoHumano/2006/home.htm) in 2006, to test whether the health outcomes, in terms of survival and toxicity, are similar to those obtained in the clinical trials of reference. In addition, the study compared the survival of patients who have completed 12 months of trastuzumab with that of patients who have received it for less time. There are no similar publications in Spain with detailed information on the cardiac toxicity and efficacy of adjuvant trastuzumab in real-world situations and with a prolonged follow-up of 15 years.

## Results

### Patient flow

**Figure 1:**
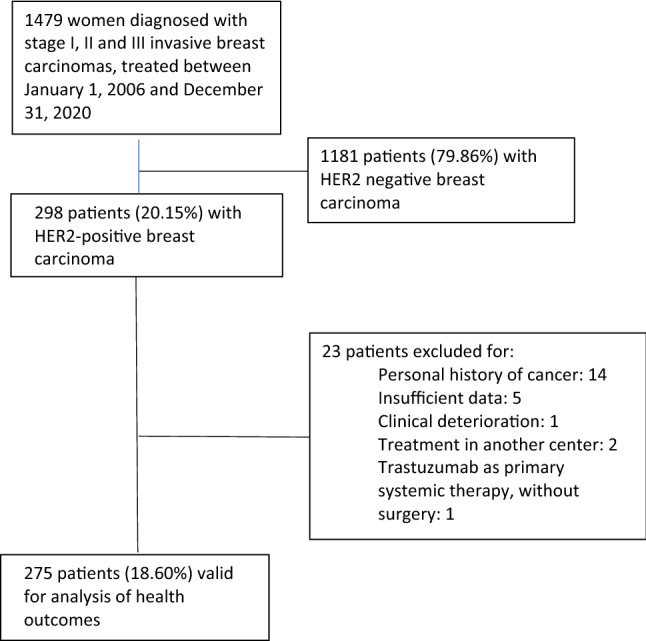
Study flowchart.

A total of 1479 medical histories of women diagnosed with breast carcinoma were reviewed, of which 298 (20.15%) were HER2-positive (Fig. 1).

### Patient characteristics

Table 1 shows the characteristics of the patients studied.

**Table 1:** Characteristics of patients, breast tumors, and treatments received.

Variable		No	%
Sex			
Female		274	99.60
Male		1	0.40
Age (median and range)	53 (26–83)		
Functional capacity^a^			
0		168	61.10
1		93	33.80
2		5	1.80
Unknown		9	3.30
Comorbidity index^b^			
0		174	63.30
1		61	22.20
2		16	5.80
3		10	3.60
4		5	1.80
Unknown		9	3.30
Body mass index			
< 25		84	30.50
25–29		71	25.80
≥ 30		65	23.60
Unknown		55	20
Arterial hypertension			
Yes		77	28
No		197	71.60
Unknown		1	0.40
Menopausal status			
Premenopausal		114	41.50%
Postmenopausal		158	57.50
Unknown		3	1.10
Histopathological type			
Ductal		259	94.20
Lobular		10	3.60
Others		1	0.40
Unknown		5	1.80
Histological grade			
G1		22	8
G2		142	51.60
G3		70	25.50
Gx		41	14.90
Extensive peritumoral vascular invasion			
Yes		60	21.80
No		176	64
Unknown		39	14.20
Tumor size in cm (median and range)	2 (0–9)		
Pathological stage			
0^c^		35	12.70
IA		99	36
IB		16	5.8
IIA		49	17.80
IIB		31	11.30
IIIA		32	11.60
IIIB		7	2.50
IIIC		4	1.50
Unknown		2	0.70
pT			
pT1mi		1	0.40
pT1a		7	2.50
pT1b		16	5.80
pT1c		63	22.90
pT2		105	38.20
pT3		8	2.90
pT4		1	0.40
pTx		2	0.70
ypT			
ypT0		33	12
ypTis		6	2.20
ypT1mi		2	0.70
ypT1a		2	0.70
ypT1b		7	2.50
ypT1c		9	3.30
ypT2		5	1.80
ypT3		5	1.80
ypT4		3	1.10
pN			
pN0		110	40
pN1mi		9	3.30
pN1		49	17.80
pN2		23	8.40
pN3		13	4.70
pNx		1	0.40
ypN			
ypN0		52	18.90
ypN1mi		3	1.10
ypN1		6	2.20
ypN2		9	3.30
Number of lymph nodes with metastases (median and range)	0 (0–21)		
Number of nodes analyzed (median and range)	6 (0–35)		
Estrogen receptors			
Positives		171	62.20
Negatives		104	37.80
Progesterone receptors			
Positives		141	51.30
Negatives		134	48.70
HER2			
Immunohistochemistry (IHC) 3+		246	89.40
IHC 2+ with gene amplification		29	10.50
Ki67 (median and range)	30 (1–100)		
Breast surgery			
Conservative		124	45.10
Mastectomy		150	54.50
Unknown		1	0.4
Axillary surgery			
Sentinel lymph node biopsy		137	49.80
Axillary lymphadenectomy		138	50.20
Radiotherapy		215	78.20
Adjuvant hormone therapy		171	62.20
Type of adjuvant hormone therapy			
Tamoxifen		92	33.50
Aromatase inhibitor (AI)		34	12.40
Tamoxifen-AI		39	14.20
Tamoxifen and goserelin		6	2.20
AI and goserelin		2	0.70
Unknown		7	2.50
Adjuvant chemotherapy		201	73.10
Neoadjuvant chemotherapy		72	26.20
Type of chemotherapy			
Anthracycline		16	5.80
Taxane		43	15.60
Anthracycline-taxane		190	69.10
Platinum-taxane		9	3.30
Others		11	4
None		1	0.40
Unknown		5	1.80
Trastuzumab-chemotherapy			
Concomitant		243	88.40
Sequential		27	9.80
Unknown		5	1.80
Trastuzumab			
Intravenous		149	54.20
Subcutaneous		122	44.40
Unknown		4	1.50
Neoadjuvant trastuzumab-pertuzumab		19	6.90

^a^ECOG Scale (East Cooperative Oncology Group).

^b^Charlson Index.

^c^Absence of post-neoadjuvant infiltrating tumor.

The median number of three-weekly cycles of trastuzumab administered was 17 (range: 1–23). Regarding trastuzumab discontinuation, 68 patients (24.70%) received 16 or fewer cycles, and 205 (74.50%) received 17 or more cycles. The most frequent causes of trastuzumab discontinuation were asymptomatic LVEF decline (54, 19.64%) and heart failure (12, 4.36%).

In the 72 patients treated with neoadjuvant systemic therapy, pathologic complete response in the breast was obtained in 39 patients (54.17%), and in the axilla in 52 patients (72.22%). Thirty-five patients achieved complete pathologic response (48.61%).

### Association between variables and the number of trastuzumab cycles

In the bivariate analysis the dependent variable “number of trastuzumab cycles” was statistically associated with age, with the way of administering chemotherapy and trastuzumab (concomitant or sequential) and with cardiotoxicity (Table 2). In the multivariate analysis, age and cardiotoxicity remained statistically significant (Table 2). For the number of trastuzumab cycles, only age (OR 0.371, 95% CI 0.152–0.903; p = 0.029) and cardiotoxicity (OR 15.02, 95% CI 7.437–30.335; p < 0.001) remain in the final multivariate model as definitively significant.

**Table 2:** Association between the number of trastuzumab cycles and predictor variables.

Predictor variables	Bivariate OR (CI95%)	p value	Multivariate OR (CI95%)	p value	Final multivariate OR after model selection	p value
Age						
≤ 65	1		1		1	
> 65	0.363 (0.190–0.692)	0.002	0.274 (0.085–0.877)	0.029	0.371 (0.152–0.903)	0.029
Menopausal status						
Premenopausal	1		1			
Postmenopausal	0.699 (0.407–1.199)	0.193	2.036 (0.746–5.559)	0.165		
Functional capacity^a^						
0	1		1			
≥ 1	0.850 (0.492–1.466)	0.558	1.006 (0.417–2.427)	0.989		
Comorbidity index^b^						
0	1		1			
≥ 1	0.814 (0.467–1.418)	0.467	1.043 (0.418–2.605)	0.928		
Body mass index						
< 25	1		1			
≥ 25	0.504 (0.265–0.958)	0.037	0.640 (0.266–1.540)	0.319		
Arterial hypertension						
Yes	1		1			
No	2.167 (1.237–3.795)	0.007	1.725 (0.650–4.580)	0.273		
Tumor size						
≤ 2 cm	1		1			
> 2 cm	0.809 (0.477–1.371)	0.430	0.875 (0.357–2.143)	0.770		
Lymph nodes with metastases						
Negative	1		1			
Positive	0.808 (0.476–1.371)	0.429	0.797 (0.319–1.989)	0.627		
Estrogen receptors						
Positives	1		1			
Negatives	0.789 (0.461–1.348)	0.385	0.994 (0.310–3.187)	0.992		
Progesterone receptors						
Positives	1		1			
Negatives	0.779 (0.461–1.316)	0.351	0.595 (0.192–1.845)	0.368		
Trastuzumab						
Adjuvant	0.824 (0.444–1.527)		0.743 (0.259–2.133)			
Neoadjuvant	1	0.583	1	0.581		
Trastuzumab-chemotherapy						
Concomitant	1		1			
Sequential	0.291 (0.129–0.656)	0.003	0.474 (0.136–1.653)	0.241		
Neoadjuvant trastuzumab-pertuzumab						
No	0.408 (0.088–1.884)		0.303 (0.031–2.965)			
Yes	1	0.251	1	0.305		
Radiotherapy						
Yes	1		1			
No	1.186 (0.622–2.261)	0.604	0.777 (0.289–2.088)	0.617		
Cardiotoxicity						
Yes	1		1		1	
No	14.235 (7.323–27.67)	< 0.001	18.78 (7.767–45.408)	< 0.001	15.02 (7.437–30.335)	0.001

Bivariate and multivariate analyses.

OR, odds ratio; CI, confidence interval.

^a^ECOG Scale (East Cooperative Oncology Group).

^b^Charlson Index.

### Association between variables and cardiotoxicity

Of all the variables none was significantly associated with cardiotoxicity in the bivariate analysis, and only the way of administering chemotherapy and trastuzumab (concomitant or sequential) and radiotherapy treatment, approached statistical significance in the multivariate analysis (Table 3). For the cardiotoxicity as a dependent variable only radiotherapy treatment showed statistical significance in the multivariate model (OR 0.0362, 95% CI 0.139–0.938; p = 0.037).

**Table 3:** Risk of trastuzumab cardiotoxicity depending on variables.

Predictor variables	Bivariate OR (CI95%)	p value	Multivariate OR (CI95%)	p value	Final multivariate OR after model selection	p value
Age						
≤ 65	1		1			
> 65	1.542 (0.777–3.061)	0.216	1.747 (0.631–4.837)	0.283		
Menopausal status						
Premenopausal	1		1			
Postmenopausal	1.230 (0.696–2.174)	0.476	0.888 (0.400–1.973)	0.771		
Functional capacity^a^						
0	1		1			
≥ 1	1.002 (0.561–1.791)	0.994	0.864 (0.386–1.932)	0.721		
Comorbidity index^b^						
0	1		1			
≥ 1	0.551 (0.203–1.560)	0.883	0.745 (0.329–1.685)	0.480		
Body mass index						
< 25	1		1			
≥ 25	1.393 (0.722–2.688)	0.323	1.147 (0.547–2.406)	0.717		
Arterial hypertension						
Yes	1		1			
No	0.715 (0.393–1.302)	0.273	0.844 (0.364–1.960)	0.694		
Tumor size						
≤ 2 cm	1		1			
> 2 cm	1.088 (0.622–1.904)	0.767	0.641 (0.290–1.413)	0.270		
Lymph nodes with metastases						
Negative	1		1			
Positive	1.489 (0.852–2.604)	0.163	2.138 (0.957–4.777)	0.064		
Estrogen receptors						
Positives	1		1			
Negatives	1.398 (0.796–2.456)	0,244	2,026 (0,716–5,736)	0,183		
Progesterone receptors						
Positives	1		1			
Negatives	1.081 (0.621–1.882)	0.784	0.800 (0.286–2.238)	0.671		
Trastuzumab						
Adjuvant	1.083 (0.579–2.023)		1.307 (0.527–3.241)			
Neoadjuvant	1	0.804	1	0.564		
Trastuzumab-chemotherapy						
Concomitant	1		1			
Sequential	2.493 (1.083–5.741)	0.032	2.772 (0.979–7.846)	0.055		
Neoadjuvant trastuzumab-pertuzumab						
No	1.483 (0.481–4.302)		0.403 (0.084–1.926)			
Yes	1	0.516	1	0.255		
Radiotherapy						
Yes	1		1		1	
No	0.493 (0.228–1.066)	0.072	0.375 (0.138–1.017)	0.054	0.0362 (0.139–0.938)	0.037

Bivariate and multivariate analyses.

OR, odds ratio; CI, confidence interval.

^a^ECOG Scale (East Cooperative Oncology Group).

^b^Charlson Index.

### Follow-up of patients and events

The median follow-up of the patients was 71 months (range: 4–194). In this period, 49 patients (17.90%) presented events that are summarized in Table 4.

**Table 4:** Events occurring in 275 patients with HER2-positive breast carcinoma.

Events	No	%
Local recurrence		
After conservative surgery	2	0.73
After mastectomy	1	0.36
Regional lymph node recurrence	3	1.09
Metastasis	25	9.16
Second infiltrating tumor		
Ipsilateral breast	1	0.36
Contralateral breast	4	1.45
Endometrial carcinoma	1	0.36
Pancreatic carcinoma	1	0.36
Gastric carcinoma	1	0.36
Colon carcinoma	1	0.36
Melanoma	1	0.36
Multiple myeloma	1	0.36
Death from		
Breast cancer	16	5.82
Heart failure	1	0.36
Ischemic heart disease	1	0.36
Pulmonary thromboembolism	1	0.36
Infection	3	1.09
Pancreatic carcinoma	1	0.36
Cause unknown	3	1.09

One patient died from heart failure in the Intensive Care Unit. She had developed cardiogenic shock during the period they were receiving adjuvant trastuzumab. Another patient died from ischemic heart disease several years after adjuvant trastuzumab had been discontinued.

### Survival analysis

**Figure 2:**
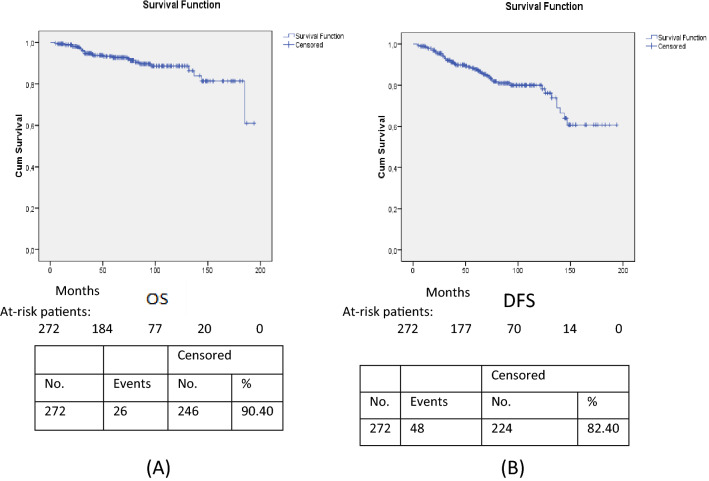
Overall (**A**) and disease-free (**B**) survival curves.

The probability of surviving after 5 years for the 275 patients was 0.93 (95% CI 0.89–0.96), at 10 years it was 0.88 (95% CI 0.84–0.93) and at 15 years 0.81 (95% CI 0.72–0.90). Five-year DFS was 0.88 (95% CI 0.83–0.92), 10-year DFS was 0.80 (95% CI 0.74–0.86), and 15-year DFS was 0.61 (95% CI 0.48–0.74). Figure 2 shows the OS and DFS curves (A, B). The median OS and DFS have not been reached.

Tables 5 and 6 present the associations observed in the bivariate and multivariate analysis between the different variables and OS and DFS.

**Table 5:** Associations between the different variables and the OS.

Predictor variables	Hazard ratio for OS					
	Bivariate HR (IC95%)	p value	Multivariate HR (CI95%)	p value	Final multivariate HR after model selection	p value
Age						
≤ 65	1		1			
> 65	3.184 (1.442–7.029)	0.004	2.208 (0.755–6.455)	0.148		
Menopausal status						
Premenopausal	1		1			
Postmenopausal	2.583 (1.042–6.405)	0.040	0.918 (0.272–3.098)	0.891		
Functional capacity^a^						
0	1		1			
≥ 1	1.070 (0.472–2.426)	0.872	0.480 (0.173–1.334)	0.158		
Comorbidity index^b^						
0	1		1			
≥ 1	1.920 (0.889–4.147)	0.097	1.422 (0.518–3.904)	0.494		
Body mass index						
< 25	1		1			
≥ 25	1.082 (0.477–2.455)	0.851	0.532 (0.202–1.402)	0.202		
Arterial hypertension						
Yes	1		1		1	
No	0.313 (0.146–0.672)	0.003	0.272 (0.098–0.758)	0.013	0.361 (0.151–0.863)	0.022
Tumor size						
≤ 2 cm	1		1			
> 2 cm	0.645 (0.292–1.422)	0.277	0.841 (0.300–2.362)	0.743		
Lymph nodes with metastases						
Negative	1		1			
Positive	1.291 (0.541–3.078)	0.573	1.642 (0.571–4.728)	0.358		
Estrogen receptors						
Positives	1		1			
Negatives	1.346 (0.630–2.873)	0.443	0.751 (0.212–2.669)	0.658		
Progesterone receptors						
Positives	1		1			
Negatives	1.396 (0.634–3.075)	0.407	1.296 (0.378–4.446)	0.680		
Trastuzumab						
Adjuvant	0.414 (0.189–0.909)	0.028	0.248 (0.090–0.684)	0.007	0.314 (0.132–0.750)	0.009
Neoadjuvant	1		1		1	
Trastuzumab-chemotherapy						
Concomitant	1		1			
Sequential	0.889 (0.292–2.704)	0.835	0.763 (0.224–2.598)	0.665		
Neoadjuvant trastuzumab-pertuzumab						
No	0.455 (0.059–3.522)	0.451	––^c^	0.979		
Yes	1					
Radiotherapy						
Yes	1		1			
No	0.754 (0.284–2.001)	0.571	1.041 (0.305–3.553)	0.949		
Number of cycles of trastuzumab						
> 16	1		1			
** ≤ 16**	1.697 (0.754–3.819)	0.201	0.650 (0.210–2.016)	0.456		
Cardiotoxicity						
Yes	2.374 (1.094–5.151)	0.029	4.224 (1.337–13.343)	0.014	2.755 (1.235–6.143)	0.013
No	1		1			

Hazard ratios from a bivariate and multivariate analysis.

HR, hazard ratio; CI, confidence interval.

^a^ECOG Scale (East Cooperative Oncology Group).

^b^Charlson Index.

^c^Highly disproportionate estimator for the size of the sample in this group.

**Table 6:** Associations between the different variables and DFS.

Predictor variables	Hazard ratio for DFS					
	Bivariate HR (IC95%)	p value	Multivariate HR (IC95%)	p value	Final multivariate HR after model selection	p value
Age						
≤ 65	1		1			
> 65	1.370 (0.701–2.679)	0.357	1.329 (0.530–3.333)	0.544		
Menopausal status						
Premenopausal	1		1			
Postmenopausal	1.227 (0.693–2.173)	0.483	0.746 (0.323–1.720)	0.491		
Functional capacity^a^						
0	1		1			
≥ 1	1.009 (0.551–1.848)	0.977	0.948 (0.440–2.039)	0.890		
Comorbidity index^b^						
0	1		1			
≥ 1	1.055 (0.571–1.949)	0.864	1.098 (0.483–2.496)	0.823		
Body mass index						
< 25	1		1			
≥ 25	1.061 (0.561–2.007)	0.855	0.682 (0.325–1.432)	0.312		
Arterial hypertension						
Yes	1		1			
No	0.769 (0.427–1.386)	0.390	0.476 (0.199–1.135)	0.094		
Tumor size						
≤ 2 cm	1		1			
> 2 cm	1.046 (0.597–1.834)	0.876	1.025 (0.482–2.180)	0.949		
Lymph nodes with metastases						
Negative	1		1			
Positive	1.297 (0.677–2.484)	0.433	1.757 (0.748–4.125)	0.196		
Estrogen receptors						
Positives	1		1			
Negatives	0.818 (0.458–1.462)	0.498	0.658 (0.238–1.823)	0.421		
Progesterone receptors						
Positives	1		1			
Negatives	0.830 (0.474–1.453)	0.515	0.996 (0.389–2.555)	0.994		
Trastuzumab						
Adjuvant	0.547 (0.298–1.004)	0.052	0.400 (0.179–0.894)	0.026	0.437 (0.213–0.899)	0.024
Neoadjuvant	1		1		1	
Trastuzumab-chemotherapy						
Concomitant	1		1			
Sequential	1.251 (0.607–2.578)	0.545	1.272 (0.534–3.031)	0.587		
Neoadjuvant trastuzumab-pertuzumab						
No	0.252 (0.075–0.851)	0.026	0.860 (0.096–7.713)	0.893		
Yes	1		1			
Radiotherapy						
Yes	1		1			
No	1.140 (0.606–2.144)	0.685	0.939 (0.379–2.327)	0.892		
Number of cycles of trastuzumab						
> 16	1		1			
≤ 16	0.764 (0.425–1.373)	0.368	0.862 (0.382–1.944)	0.728		
Cardiotoxicity						
Yes	1.334 (0.726–2.450)	0.353	1.900 (0.810–4.456)	0.140		
No	1		1			

Hazard ratios from a bivariate and multivariate analysis.

HR, hazard ratio; CI, confidence interval.

^a^ECOG Scale (East Cooperative Oncology Group).

^b^Charlson Index.

In the multivariate model, hypertension (HR 0.361, 95% CI 0.151–0.863, p = 0.022), neoadjuvant treatment (HR 0.314, 95% CI 0.132–0.750, p = 0.009), and cardiotoxicity (HR 2.755, 95% CI 1.235–6.143, p = 0.013) maintained significant association with OS. Only the neoadjuvant treatment maintained a significant association with DFS (HR 0.437, 95% CI 0.213–0.899, p = 0.024).

## Discussion

The results presented confirm the effectiveness of trastuzumab in the real world, with OS and DFS that can be considered comparable to those of clinical trials^6–8^. In the *Early Breast Cancer Trialists’' Collaborative group* (EBCTCG) meta-analysis^17^, with a median follow-up of 10.7 years, 26.60% of patients had recurrence of HER2 breast cancer and 19.70% died. For our patients, with a shorter follow-up, 6 years, 11.34% had recurrence of breast cancer and 9.44% died. The real-world effectiveness of trastuzumab is also demonstrated when it was administered with neoadjuvant intent, with pathologic complete response in 48.61% of cases, better or similar to that obtained in trials involving chemotherapy and trastuzumab^18–20^.

The way of measuring cardiac toxicity, which was different for each study, means that the results are not entirely comparable. Some 18.6% of participants in the treatment arm with doxorubicin and cyclophosphamide followed by docetaxel plus trastuzumab, in the BCIRG 006 trial (follow-up 65 months)^8^, 14% in the chemotherapy plus trastuzumab treatment arm of the NSABP-31 trial (follow-up 24 months)^21^, and 7.08% in the chemotherapy arm followed by 1 year of trastuzumab in the HERA trial (follow-up 12 months)^6,22^, had significant decreases in LVEF. The percentage of grade 3 and 4 heart failure was 4.5% in a meta-analysis of five trastuzumab trials^23^. Our cardiotoxicity results were an asymptomatic decline in LVEF in 19.64% and heart failure in 4.36%. Cardiotoxicity was the main reason for discontinuing trastuzumab, with 24.70% receiving 16 or fewer cycles.

In our study, only having received radiotherapy was associated with an increased risk of cardiotoxicity, without detecting, as in other studies^15^, any association with age, arterial hypertension or obesity. The risk of cardiotoxicity associated with radiation received by the heart, and administration of potentially cardiotoxic trastuzumab is unclear. It is important to highlight that in an analysis of 1503 patients treated with adjuvant radiation in the randomized NCCTG N9831 trial, no increased cardiotoxicity was observed, even in patients treated with concomitant trastuzumab and radiotherapy^22^.

In an attempt to identify the variables associated with a lower number of trastuzumab cycles—being over 65 years of age, and suffering from cardiotoxicity showed significant odds ratios in the multivariable analysis, so we can confirm the association between not completing the 12 months of trastuzumab and age, and cardiotoxicity. Of the women studied, 17.8% were older than 65 years. In real-world studies, these older patients specifically are not well represented in clinical trials, and patients who are poorly represented experience worse outcomes than those who are well represented^23^.

One of the consequences of trastuzumab cardiotoxicity is the obligation to discontinue treatment, causing patients to be unable to receive all 17–18 cycles in the 12 months of treatment. In our real world, although patients received a median of 17 cycles, the 68 (24.70%) who received 16 or fewer cycles did not have a worse OS than those who received more than 16 cycles. These results are at odds with the systematic review of trials on de-escalation in the duration of adjuvant trastuzumab therapy^14^, where a shorter duration of adjuvant trastuzumab is associated with significantly worse DFS and OS. Other studies and meta-analyses^12,24^, however, have shown non-inferiority of 6 months compared to 12 months. These results should be interpreted with caution, as they are based primarily on the Persephone trial^12^, an older trial that employed chemotherapy-trastuzumab regimens considered nonstandard (taxane-free anthracyclines in 90% and sequential trastuzumab in 53% of cases), in a relatively low-risk population (59% of node-negative patients).

In the case of adjuvant treatment of early breast cancer with trastuzumab, short- and long-term cardiovascular side effects become crucially important in health outcomes^25^ and this is what we observed also in our real-world study, as patients who did not develop cardiotoxicity, and who do not have hypertension, had better OS than those who did have heart failure or significant decline in LVEF and hypertension. Other authors^26^ have already established the consensus that patients who do not complete adjuvant trastuzumab because of cardiotoxicity have a significantly increased risk of relapse and death from breast cancer. These findings also support 1 year of adjuvant trastuzumab^26^.

The use of trastuzumab with chemotherapy in the neoadjuvant setting results in a higher rate of complete disease responses, but the effects on DFS and OS are unclear compared to adjuvant treatment. One study supports early initiation of neoadjuvant trastuzumab, especially in patients with hormone-resistant and HER2-positive tumors^27^, so we cannot give a plausible explanation for our findings of worse OS and DFS in patients treated in the neoadjuvant setting.

This study was a retrospective observational evaluation of data intended to assess the effectiveness of adjuvant trastuzumab in the real world and is therefore subject to limitations. There may be unmeasured confounders unequally distributed among the groups. There may also be uncaptured patient comorbidities. Another limitation is the need for longer follow-up, as patients with hormone-sensitive breast cancer, even if they are HER2+, may have late recurrences.

There are no similar publications in Spain with detailed data on the effectiveness and cardiac toxicity of adjuvant trastuzumab in the real world and with an extended follow-up of 15 years. For this reason, the results presented here constitute valuable information with which other centers can make comparisons.

In conclusion, our results confirm the significance of the use of trastuzumab in the adjuvant treatment of early-stage breast cancer in the real world, with survival comparable to those of clinical trials. In the real world, factors such as age, hypertension, radiotherapy, neoadjuvant treatment and cardiotoxicity should be taken into consideration to optimize outcomes.

## Methods

This is an observational, descriptive, retrospective, single-center, study on a medicinal product for human use. It was carried out at the Medical Oncology Department of a university hospital. All cases treated since the official approval of trastuzumab in 2006 were selected. Patients were identified from the Medical Oncology department’s database, and data related to patients and treatment received were retrospectively collected from Medical Oncology’s medical records. All experimental protocols were approved by the Cádiz Research Ethics Committee.

Patients with breast cancer undergoing surgical treatment with a pathology result showing HER2-positive infiltrating carcinoma were included. Those suitable for inclusion in the study had to have been treated with trastuzumab, either alone or with chemotherapy and/or hormone therapy, between 1 January 2006 and 31 December 2020, and followed up until 1 September 2021.

Female and male patients with HER2-positive infiltrating breast carcinoma (immunohistochemical score 3+ and 2+ with gene amplification), confirmed by histology, at disease stage I, II and III were included. Patients could have received adjuvant or neoadjuvant systemic treatment with hormone therapy, chemotherapy, or neither. Patients who received systemic neoadjuvant therapy were treated with chemotherapy plus trastuzumab, or plus trastuzumab and pertuzumab prior to surgery. These patients did not receive adjuvant chemotherapy but they did receive adjuvant trastuzumab with a goal of 12 months of treatment. Cases of carcinoma in situ, without infiltrating component and patients with metastases were excluded.

Age, sex, functional capacity (measured by the *ECOG* scale)^28^, menopausal status, comorbidity (measured by the Charlson scale)^29^, body mass index and arterial hypertension were the patient-related independent variables.

The independent variables related to the breast tumor were: tumor size at definitive surgery, disease stage (*American Joint Committee on Cance*^*r*^ 8th edition*)*, histologic grade, histologic type, extensive peritumoral vascular invasion, estrogen and progesterone receptor status (considered positive if immunohistochemical expression was equal to or greater than 1%), HER2 (positive if immunohistochemical score was 3+ or 2+ with gene amplification by in situ hybridization), Ki67 proliferative index, number of axillary nodes with metastases, number of total axillary nodes analyzed.

The independent variables related to treatment were: type of surgery, radiotherapy, type of adjuvant hormonal treatment, type of adjuvant chemotherapy, route of administration of trastuzumab and form of administration of chemotherapy-trastuzumab (concomitant or sequential). Weekly trastuzumab was administered intravenously at a starting dose of 4 mg/kg of body weight and maintenance dose of 2 mg/kg for 12 months. Trastuzumab was administered three times a week either intravenously at a starting dose of 8 mg/kg and maintenance dose of 6 mg/kg, or subcutaneously at a dose of 600 mg, both also for 12 months.

The independent variables related to safety were cardiotoxicity (subclassified into symptomatic heart failure and asymptomatic LVEF decline), trastuzumab discontinuation (patients were classified into those who had completed the 17–18 three-weekly cycles of trastuzumab treatment and those who have not completed treatment with ≤ 16 cycles), and the reason trastuzumab was discontinued. In case of weekly trastuzumab administration, 3 weeks was considered to be 1 cycle. A baseline LVEF of at least 50% was required to initiate trastuzumab treatment and the test was repeated every 3 months during the year of treatment. Trastuzumab was discontinued if symptomatic heart failure or an asymptomatic decrease in LVEF of 15 points, or 10 points if this put the level below 50%, occurred. In cases of asymptomatic LVEF decline, angiotensin-converting enzyme inhibitors and beta-blockers were prescribed at low doses and repeated on a month-by-month basis. If levels recovered, trastuzumab was reintroduced.

The efficacy dependent variables analyzed were overall survival and disease-free survival^30^.

A descriptive analysis of the variables (absolute and relative frequency, mean, median and standard deviation) was made. For the comparison of qualitative variables, the chi-square test with Fisher’s correction was applied and, for quantitative variables, the Student’s t-test.

The influence of independent variables on the number of trastuzumab cycles administered was measured using binary logistic regression, using odds ratio (OR) for risk estimation with a corresponding CI of 95%.

Logistic regression analysis with an independent variable was performed to calculate the odds ratio (OR) of each variable in relation to trastuzumab cardiotoxicity.

The Kaplan Meier method and the Log-Rank test for the comparison of survival curves were used to calculate survival. Cox regression with an independent variable was used to calculate the hazard ratio (HR).

Landmark analysis was carried out to monitor immortal time bias in the survival analysis among subgroups with ≤ 16 cycles and 17–18 cycles of trastuzumab. The landmark time is established as the duration of time that it would take to complete 16 cycles. Any patients experiencing event (DFS or OS) prior to this landmark time were dropped from the analytical sample. The groups (≤ 16 cycles versus 17–18 cycles) were defined, using only patients in the group without events prior to the landmark time. The survival analysis was conducted as usual, with t0 set equal to the landmark time.

The SPSS program, version 21, was used for statistical analysis of the data. In the statistical analysis, p < 0.05 was considered to indicate statistical significance.

### Ethical considerations

The work described was carried out in accordance with the Code of Ethics of the World Medical Association (Declaration of Helsinki) for experiments on human subjects. All experimental protocols were approved by the Cádiz Research Ethics Committee (code 152.21), which authorized the waiver of informed consent.

## Data Availability

The datasets used and/or analyzed during the current study available from the corresponding author on reasonable request.
